# Species in the *Cryptococcus gattii* Complex Differ in Capsule and Cell Size following Growth under Capsule-Inducing Conditions

**DOI:** 10.1128/mSphere.00350-16

**Published:** 2016-12-28

**Authors:** Kenya E. Fernandes, Christine Dwyer, Leona T. Campbell, Dee A. Carter

**Affiliations:** School of Life and Environmental Sciences, University of Sydney, Sydney, New South Wales, Australia; Carnegie Mellon University

**Keywords:** *Cryptococcus gattii*, capsule, cryptic species, osmotic stress, oxidative stress, thermotolerance, yeasts

## Abstract

*Cryptococcus gattii* causes invasive fungal infections that have been increasing in incidence and global distribution in recent years. The major molecular genotypes of *C. gattii* that were previously classified as VGI to VGIV have recently been described as four new species: *C. gattii* (VGI), *C. deuterogattii* (VGII), *C. bacillisporus* (VGIII), and *C. tetragattii* (VGIV). The main driver for their classification has been phylogeny, and phenotypic diversity has not yet been extensively characterized. This study examines variation in attributes related to virulence and pathogenicity, including capsule thickness, cell size, tolerance to temperature, oxidative and osmotic stress, and cell wall integrity. A capsule induction agar using diluted Sabouraud medium revealed significant differences in capsule and cell size across the *C. gattii* species complex and produced irregularly shaped elongated cells in a number of strains. *C. gattii*/VGI strains possessed the largest capsules of all species but had smaller cells, while *C. deuterogattii*/VGII strains possessed the largest cells of all species but had smaller capsules. Overall thermotolerance was highest in *C. deuterogattii*/VGII strains, while a number of *C. bacillisporus*/VGIII, and *C. tetragattii*/VGIV strains had substantially reduced growth at 37°C. There was no significant difference among species in their tolerances to oxidative or osmotic stresses, and there was no evidence for defects in cell wall integrity in strains producing irregular cells. These data support the division of the *C. gattii* species complex into distinctly identified species and suggest underlying reasons for their differences in virulence, epidemiology, and host preference.

**IMPORTANCE** Infections with the fungal pathogen *Cryptococcus gattii* have been increasing in recent years. Recently, four different species have been described within *C. gattii*, which correspond to four previously known molecular genotypes (VGI to VGIV). Examining traits related to infection and disease is important for determining whether these different species have clinical relevance. This study examined variation in attributes that are important for infecting and surviving in the host, including tolerance to various stresses, yeast cell size, and the amount of polysaccharide capsule that covers the cell. The cell size and capsule size were significantly different and inversely correlated across the species. Thermotolerance was highest in *C. deuterogattii* (VGII), the only species known to cause outbreaks, while most strains of the species* C. bacillisporus* (VGIII) and *C. tetragattii* (VGIV) grew poorly at 37°C. These findings argue for increased acceptance of the new species and may be useful for informing diagnosis and prognosis in clinical infection.

## Abstract (precis)

Infections with the fungal pathogen *Cryptococcus gattii* have been increasing in recent years. Recently, four different species have been described within *C. gattii*, which correspond to four previously known molecular genotypes (VGI to VGIV). Examining traits related to infection and disease is important for determining whether these different species have clinical relevance. This study examined variation in attributes that are important for infecting and surviving in the host, including tolerance to various stresses, yeast cell size, and the amount of polysaccharide capsule that covers the cell. The cell size and capsule size were significantly different and inversely correlated across the species. Thermotolerance was highest in *C. deuterogattii* (VGII), the only species known to cause outbreaks, while most strains of the species* C. bacillisporus* (VGIII) and *C. tetragattii* (VGIV) grew poorly at 37°C. These findings argue for increased acceptance of the new species and may be useful for informing diagnosis and prognosis in clinical infection.

## INTRODUCTION

*Cryptococcus gattii* is a pathogenic yeast that has seen an expanded geographic distribution in recent years, causing increased numbers of fatal infections in humans and other animals (1–3). Infection is acquired from the environment via the inhalation of spores or desiccated yeast cells into the alveolar spaces of the lung (4, 5). From the lung, *Cryptococcus* is capable of disseminating to any organ of the body with a proclivity for the central nervous system (6). As such, the most frequent and severe presentation of cryptococcosis is meningoencephalitis (7). Unlike its sibling species *Cryptococcus neoformans*, which is a major AIDS pathogen, *C. gattii* tends to infect apparently healthy hosts (8, 9), although HIV-related cases have been documented (10–12).

*C. gattii* has recently been redescribed as a species complex comprising multiple independent species (13). These species were previously considered four distinct molecular genotypes, VGI to VGIV, divided by various molecular methods (14). *Cryptococcus gattii* (previously VGI) and *Cryptococcus deuterogattii* (previously VGII) are the most frequently encountered and widely distributed. *C. gattii*/VGI accounts for most Australian isolates, while *C. deuterogattii*/VGII is common in South America and is responsible for a large, ongoing outbreak in the American Pacific Northwest (15–17). *Cryptococcus bacillisporus* (previously VGIII) is prevalent in the Ibero American countries and has been found in India and the United States, while *Cryptococcus tetragattii* (previously VGIV) has been reported in India, Africa, and Central America (14, 15, 18). A fifth species, *Cryptococcus decagattii*, has been proposed for a phylogenetically distinct cluster within the species complex, but currently it consists of only 5 isolates (13).

Despite their elevation to species status, there is relatively little known about the phenotypic differences among these genetically diverse clades. This warrants further investigation, particularly for attributes associated with virulence and pathogenicity that may influence disease progression and treatment (19). Pathogenic *Cryptococcus* species possess a range of virulence factors and physiological attributes that enable them to survive inside the human host. The latter includes the ability to grow at 37°C and the capacity to tolerate oxidative and other stresses (20), which have been found *in vitro* to correlate with virulence (21). Previous studies have found significant differences in antifungal susceptibility with *C. deuterogattii*/VGII being the least susceptible to azole antifungal agents (22); however, to date, most studies have focused on individual isolates, and variation among the species/genotypes has not been ascertained in detail for many phenotypes.

Of the major virulence factors in *Cryptococcus*, possession of a highly hydrophilic extracellular capsule is considered the most significant. The capsule is mainly composed of two complex branching polysaccharides, glucuronoxylomannan and glucuronoxylomannogalactan (23, 24), and performs many protective functions, including inhibiting ingestion by phagocytes, acting as a sink for reactive oxygen species, and facilitating immune evasion and survival within the host (25–27). Studies of acapsular mutants in animal models have shown these to have a profound reduction in virulence (25). However, data on the relationship between the extent of capsulation and virulence are conflicting, with some studies reporting that highly encapsulated strains are more virulent (28), while others report the inverse (29).

The size of the capsule is highly dynamic, and a dramatic increase in capsule size is induced during mammalian infection, stimulated by stresses imposed by the host environment (30). In addition to capsule enlargement, the formation of yeast body size variants in the form of giant cells >25 μm in diameter and micro cells <1 μm in diameter (31, 32), as well as the release of extracellular capsule have been observed and are thought to play a role in infection. When grown *in vitro* on rich media commonly used in research laboratories, such as Sabouraud agar, the size of the capsule is relatively small. However, capsule can be induced by employing conditions that simulate the host environment, including mammalian body temperature, reduced nutrients, and elevated CO_2_ concentration (27). Various capsule-inducing media have been reported for use in *C. neoformans*, but these have not yet been used in the study of species in the *C. gattii* complex.

The aim of the current study was to investigate whether virulence-related attributes vary among the newly described species of *C. gattii*, including capsule size and tolerance to heat and oxidative stress. Using a low-nutrient capsule induction medium, we show differences in cell and capsule size among the species, with *C. gattii*/VGI having significantly larger capsules than all other species but smaller cells and *C. deuterogattii*/VGII having smaller capsules but significantly larger cells. Growth under capsule-inducing conditions led to a number of strains producing irregular cells that did not appear to be due to cell membrane or cell wall defects, as all strains were equally affected by the presence of detergent, and the response to salt, while variable, did not correlate with the presence of irregular cells. *C. deuterogattii*/VGII was more tolerant overall to elevated temperature than the other species, and the growth of a number of *C. bacillisporus*/VGIII and *C. tetragattii*/VGIV strains was substantially reduced at 37°C. There was no significant difference among species in their response to oxidative stress. Overall we show substantial and reproducible *in vitro* differences in virulence-related attributes among the species.

## RESULTS

### CIM-20 reliably induces capsule enlargement across species in the *C. gattii* complex.

**FIG 1:**
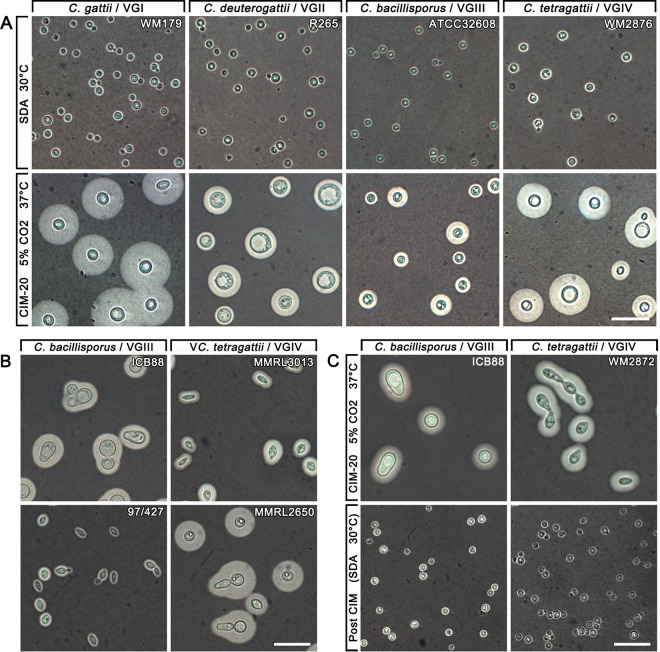
Capsule enlargement and morphologically irregular cells following growth under capsule-inducing conditions. (A) Indian ink preparations of representative strains from each species showing capsule enlargement when grown on CIM with 5% CO_2_ at 37°C for 5 days compared to growth on SDA at 30°C for 5 days. (B) Growth on CIM-20 with 5% CO_2_ at 37°C induced irregular, elongated cell shapes, particularly in *C. bacillisporus*/VGIII and *C. tetragattii*/VGIV strains. (C) Normal growth resumed following subculture on SDA at 30°C. Bars = 20 µm.

In *C. neoformans*, capsule induction uses Sabouraud medium diluted 10-fold in 50 mM morpholinepropanesulfonic acid (MOPS) (CIM-10) (27); however, this did not reliably induce capsule in the *C. gattii* complex strains. Different media were therefore used, including Dulbecco’s modified Eagle medium (DMEM) and Sabouraud dextrose agar (SDA) diluted 20-fold (CIM-20) or 50-fold (CIM-50) in 50 mM MOPS. These media were incubated with 5% CO_2_ at 37°C for 5 days. Capsule enlargement was tested on the range of induction media and standard SDA at 30°C to compare with baseline capsule production. Capsule thickness and cell diameter were measured across the various media after 5 days of growth (see Fig. S1A to S1C in the supplemental material). Cells grown on SDA without CO_2_ at 30°C had very little capsule production. Cells grown on CIM-50 grew very poorly beyond the primary inoculum site, and this medium was excluded from further testing. CIM-20 induced the largest capsules and large cell diameters while still allowing healthy cell growth, and it worked consistently as a growth medium for the different species, allowing substantial and reproducible differences to be seen (Fig. 1A). Therefore, CIM-20 was chosen as the growth medium for all subsequent analyses.

10.1128/mSphere.00350-16.1 Figure S1  Optimization of capsule enlargement medium in *C. gattii*/VGI strain WM179**.** (A) Indian ink preparations of *C. gattii*/VGI strain WM179 showing capsule size when grown on a range of induction media with 5% CO_2_ at 37°C for 5 days compared to growth on SDA at 30°C for 5 days. Scale bar = 20 µm. (B and C) Capsule thickness (B) and yeast cell diameter (C) of WM179 cells when grown on a range of induction media. Values are means (horizontal bars) ± 95% confidence intervals (error bars) (*n* = 25). Download Figure S1, TIF file, 1 MB. Copyright © 2016 Fernandes et al. 2016 Fernandes et al. This content is distributed under the terms of the Creative Commons Attribution 4.0 International license.

### Capsule-inducing conditions cause morphologically irregular cells in some *C. gattii*/VGI, *C. bacillisporus*/VGIII, and *C. tetragattii*/VGIV strains.

More than half of the *C. gattii*/VGI, *C. bacillisporus*/VGIII, and *C. tetragattii*/VGIV strains produced some elongated and irregularly shaped cells when grown on CIM-20 (Table 1; Fig. 1B). *C. bacillisporus/*VGIII had the greatest proportion of irregular cells, with more than 10% of cells having an irregular phenotype in 7 of the 14 strains and reaching >75% of cells in strain DUMC140.97. In *C. gattii*/VGI and *C. tetragattii*/VGIV, irregular cells were seen in a number of strains, but they were generally a minority (<10%) of all cells. No irregular cells were observed in any of the *C. deuterogattii*/VGII strains. As the irregular shapes made it difficult to accurately measure cell and capsule sizes, strains where >10% of the cells were irregular were excluded from measurements. When strains containing irregular cells were subcultured and grown under standard conditions on SDA, all cells reverted to their normal phenotype, indicating that cell irregularity is induced by the conditions used to induce capsule and is not a stable feature (Fig. 1C).

**TABLE 1:** Strains used in this study with details of cell and capsule sizes^a^

Species/genotype and strain	*MAT*	Source	Geographic origin	Cell diameter (µm)		Capsule thickness (µm)		Volume ratio		Irregular cells (%)^c^	Giant cells^d^
				Mean	SD^b^	Mean	SD^b^	Mean	SD^b^		
*C. gattii*/VGI											
2005/215	α	Clinical	France	6.1	1.0	6.1	1.0	29.3	9.0		
327/99	α	Veterinary	Australia	6.6	1.0	8.5	1.0	48.6	14.7		
571_094	α	Veterinary	Australia	6.6	0.7	7.8	0.8	39.1	8.2	<10	
E566	**a**	Environmental	Australia	6.7	0.6	5.3	0.4	17.6	3.6	<10	
Env316	α	Environmental	Australia	6.2	0.9	5.7	1.0	23.3	6.1	<10	
Env71	α	Environmental	Australia	7.1	0.7	5.2	0.5	15.6	2.8	<10	
F2863	α	Veterinary	Canada	5.7	0.7	5.2	0.5	24.0	7.6		
NT-2	α	Clinical	Australia	6.5	0.8	3.0	1.0	7.7	3.7	<10	
NT-10	α	Clinical	Australia	7.1	0.9	5.0	0.8	14.7	3.3		
PNG14	**a**	Clinical	Papua New Guinea							**>10**	
PNG19	**a**	Clinical	Papua New Guinea	9.3	1.0	6.6	1.1	15.0	4.8		
PNG27	**a**	Clinical	Papua New Guinea	7.6	1.0	6.6	1.9	22.0	11.0	<10	
R794	α	Clinical	Canada							**>25**	
V12	**a**	Environmental	Australia	6.2	0.7	6.6	0.7	31.1	6.9	<10	
V13	α	Veterinary	Australia	8.2	1.4	2.8	0.7	5.1	2.0		
V15/571_103	α	Veterinary	Australia	6.6	1.1	7.9	1.5	39.4	9.6	<10	
V24/571_134	α	Veterinary	Australia	8.2	0.6	5.7	0.4	14.0	2.7		
WM1272	α	Clinical	Papua New Guinea	6.2	0.8	5.7	1.3	24.5	8.5		
WM179	α	Clinical	Australia	7.9	1.0	10.4	1.6	49.2	12.3	<10	

All strains				7.0	0.9	6.1	3.3	24.7	163.5	58	0

*C*. *deuterogattii*/VGII											
14 1431	α	Veterinary	Australia	8.1	1.1	3.2	0.7	6.0	2.1		
14 1433	α	Veterinary	Australia	10.1	2.2	3.1	1.2	4.5	1.7		✓
2001/935-1	α	Clinical	Senegal	6.5	0.8	1.8	0.4	4.0	1.2		
2003/125	α	Clinical	France	7.0	0.6	3.0	0.6	6.4	1.7		
2004/335	α	Clinical	French Guyana	6.4	1.0	2.5	0.8	6.6	4.2		
93/980	α	Clinical	France	10.8	2.1	2.0	0.5	2.7	0.8		✓
97/170	α	Clinical	French Guyana	6.3	0.9	1.8	0.4	4.1	1.4		
98/1037-2	α	Clinical	France	5.8	0.6	1.9	0.3	4.8	1.3		
CBS1930	α	Veterinary	Aruba	9.3	1.8	2.8	0.8	4.5	1.8		
ICB182	α	Clinical	Brazil	7.8	2.2	1.8	0.6	3.7	1.8		
ICB184	α	Environmental	Brazil	8.8	1.3	1.7	0.4	2.9	0.8		
ICB97	α	Clinical	Brazil	10.1	2.0	3.4	1.2	5.2	2.5		
LA499	α	Clinical	Columbia	9.5	2.1	2.8	0.6	4.5	2.0		
NT-8	α	Clinical	Australia	7.7	1.0	2.0	0.6	3.7	1.1		
R265	α	Clinical	Canada	10.1	2.0	3.4	1.2	5.2	2.0		✓
RB31	α	Clinical	Canada	10.2	1.3	3.1	1.3	4.5	2.5		
RDH-9	α	Clinical	Australia	7.2	1.4	2.5	0.6	5.3	1.9		
V5/571_063	α	Veterinary	Australia	10.4	0.9	3.3	0.9	4.5	1.6		
WM178	α	Clinical	Australia	8.3	0.9	4.0	0.7	7.9	2.2		

All strains				8.4	2.5	2.6	0.5	4.8	1.5	0	16

*C. bacillisporus*/VGIII											
94/943-7	α	Clinical	Mexico	6.9	1.0	6.1	1.3	22.6	8.9		
97/427	α	Clinical	Mexico							**>10**	
ATCC 32608	α	Clinical	United States	8.0	1.9	2.4	0.9	4.7	2.6		✓
B13C	α	Clinical	Asia	5.2	0.5	2.1	0.3	6.0	1.8	<10	
CDCB4546/JH1741	**a**	Clinical		5.3	0.5	2.1	0.2	5.7	1.0		
DUMC140.97	α	Clinical	Columbia							**>75**	
ICB88	α	Clinical	Brazil							**>10**	
NIH184	α	Clinical	Australia	7.2	1.0	1.3	0.3	2.5	0.5		
NIH312/JH826	α	Clinical								**>25**	
PNG30	α	Clinical	Papua New Guinea	7.5	0.7	3.6	0.5	7.9	1.7		
PNG34	α	Clinical	Papua New Guinea							**>50**	
V28/571_169	α	Veterinary	Australia	6.2	0.9	5.1	0.8	19.5	5.9		
VBP62270	α	Veterinary	Australia							**>25**	
WM161	α	Environmental	United States							**>25**	

All strains				6.6	1.0	3.2	2.7	9.8	53.2	57	7

*C. tetragattii*/VGIV											
B+201				7.6	1.2	5.0	1.2	13.2	4.1	<10	
B5742/106.97	α	Clinical	India	4.9	1.3	2.7	0.6	10.6	4.7	<10	
B5748/107.97	α	Clinical	India	7.9	1.0	3.5	0.4	7.0	1.3		
M250	α	Clinical	Malawi	7.7	1.7	5.9	1.8	19.4	10.8	<10	
M391	α	Clinical	Botswana	4.3	0.6	2.6	0.6	11.1	4.6	<10	
MMRL2650		Clinical	India							**>10**	
MMRL2651	α	Clinical	Botswana	7.8	1.8	6.5	1.7	20.1	6.3		
MMRL2879	α	Clinical	Botswana	8.0	1.4	3.4	0.6	6.7	1.8		✓
MMRL2924	α	Clinical	Botswana	6.4	1.4	4.4	0.9	14.8	5.6		
MMRL2933	α	Clinical	Botswana	5.7	1.4	3.4	1.4	10.6	4.7	<10	
MMRL2980	α	Clinical	Botswana							**>10**	
MMRL3013	α	Clinical	Botswana	6.7	1.3	5.0	0.7	16.5	4.9	<10	
WM04.20/M772055		Clinical	South Africa	9.0	1.1	3.4	0.9	6.0	2.7		
WM2579/M27056				8.9	1.3	7.6	1.5	21.5	8.0		
WM2872	α	Clinical	Botswana							**>50**	✓
WM2876/V00869				6.8	2.5	3.9	1.3	11.2	5.2		✓
WM779	α	Veterinary	South Africa							**>25**	
WM780/V00709				7.7	0.6	4.5	0.4	10.4	1.8		

All strains				7.1	1.8	4.4	2.0	12.8	23.5	56	17

a Details of cell and capsule sizes and total cell/yeast cell volume ratio (mean and standard deviation [SD]), the percentage of irregular cells, and the presence/absence of giant cells are shown for individual strains.

b For the species/genotype overall (all strains), the variance is shown.

c The percentages of irregular cells are shown for individual strains. Strains where >10% cells were irregular are indicated in boldface type; these values were excluded from capsule and cell size analysis. For the species/genotype overall (all strains), the total percentage of isolates with irregular cells is shown.

d The presence/absence of giant cells is shown for individual strains. The presence of giant cells in an individual strain is indicated by a checkmark. For the species/genotype overall (all strains), the total percentage of isolates with giant cells is shown.

### *C. gattii* complex species differ in induced capsule and cell size.

**FIG 2:**
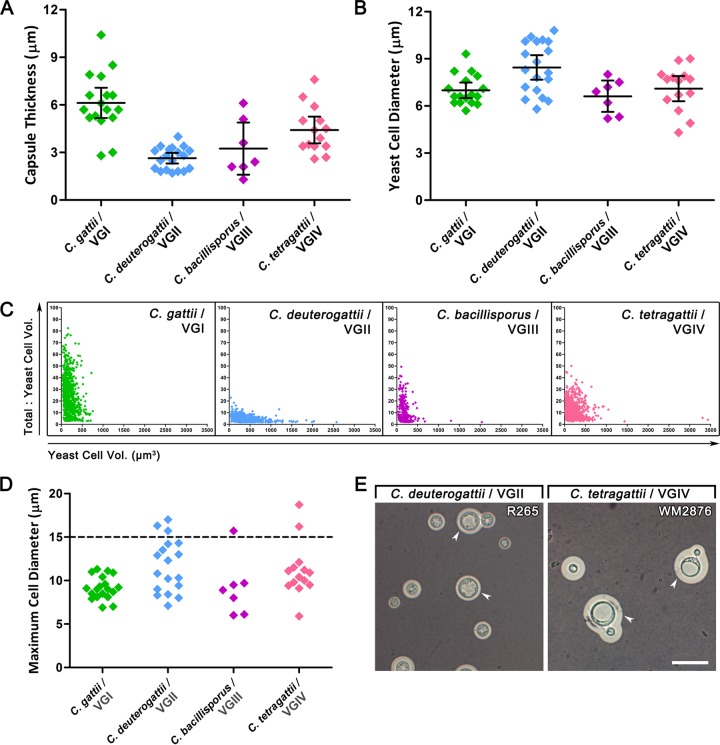
Cell diameter and capsule thickness across species in the *C. gattii* complex after growth on CIM-20. (A and B) Capsule thickness (A) and yeast cell diameter (B) of isolates from each species grown on CIM-20 with 5% CO_2_ at 37°C for 5 days. Each symbol represents the average value for 50 cells measured for a single isolate. Values are means (horizontal bars) ± 95% confidence intervals (error bars) for *C. gattii*/VGI (*n* = 17), *C. deuterogattii*/VGII (*n* = 19), *C. bacillisporus*/VGIII (*n* = 7), and *C. tetragattii*/VGIV (*n* = 14) strains. (C) Average yeast cell volume plotted against the average total cell/yeast cell volume ratio of 3,000 individual cells across all four species. Cells sitting further up the *y* axis possess larger relative capsular volume, while cells sitting further along the *x* axis possess larger cell volumes. (D) Maximum cell diameter measured across 50 cells for each isolate. *C. deuterogattii*/VGII, *C. bacillisporus*/VGIII, and *C. tetragattii*/VGIV isolates infrequently exhibited giant cells greater than 15 µm in diameter. (E) Indian ink preparations of representative strains from *C. deuterogattii*/VGII and *C. tetragattii*/VGIV showing giant cells indicated by white arrowheads. Bar = 30 µm.

Capsule thickness and yeast cell diameter for the different species and strains were examined after growth on CIM-20 with 5% CO_2_ at 37°C for 5 days, and the mean and variation were compared for the different species (Table 1; Fig. 2A and B). The average capsule thickness of *C. gattii*/VGI was significantly larger than that of *C. deuterogattii*/VGII (*P* < 0.0001), *C. bacillisporus*/VGIII (*P* = 0.0044), and *C. tetragattii*/VGIV (*P* = 0.0079). *C. tetragattii*/VGIV in turn had a significantly larger average capsule thickness than *C. deuterogattii*/VGII (*P* = 0.0006), but this was not significantly different from that of *C. bacillisporus*/VGIII. Although they had the smallest average capsule thickness, *C. deuterogattii*/VGII had a significantly larger average yeast cell diameter than *C. gattii*/VGI (*P* = 0.0024), *C. bacillisporus*/VGIII (*P* = 0.0044), and *C. tetragattii*/VGIV (*P* = 0.0158), which were not significantly different from each other. F test analysis found that capsule thickness was considerably less variable in *C. deuterogattii*/VGII than in *C. gattii*/VGI (*P* = 0.0001), *C. bacillisporus*/VGIII (*P* = 0.0018), and *C. tetragattii*/VGIV (*P* = 0.0044), but there was no significant difference among the species in the variability of yeast cell diameter.

### Capsule and cell volume measurements for individual cells group in a species-specific manner.

Given that capsule thickness and yeast cell diameter varied so widely within species when averaged across strains, we used a plot of 3,000 measurements encompassing 50 cells from each strain to explore the relationship between capsule production and yeast cell size in individual cells and the distribution of these measurements among the species. This is illustrated in Fig. 2C where the *y* axis shows the relative volume of capsule for each cell independent of the yeast cell volume, which has been standardized to 1, while the *x* axis shows the corresponding yeast cell volume. Across all species, cells with the largest relative capsule volumes possess the smallest yeast cell volumes and vice versa, indicating that capsule production does not scale up with the size of the cell. In particular, *C. gattii*/VGI cells have small yeast cell volumes with large relative capsule volumes, reaching approximately 80 times that of cell volume for some cells, while *C. deuterogattii*/VGII cells have the largest range in yeast cell volumes with consistently smaller relative capsule volumes. The majority of *C. bacillisporus*/VGIII and *C. tetragattii*/VGIV cells cluster closer to the origin with relatively smaller capsule and cellular volumes.

### Occasional giant cells are observed following growth under capsule-inducing conditions.

The typical size of cryptococcal cells grown *in vitro* is reported to be 4 to 10 µm (7). Cells with a yeast cell diameter greater than 15 µm have been defined as titan cells, and these cells can reach sizes of up to 100 µm (33). However, true titan cells possess additional defining characteristics such as increased DNA content and increased vacuole formation, and as these properties were not measured in this study, cells with a yeast cell diameter greater than 15 µm are referred to as giant cells. Giant cells were observed across species in the *C. gattii* complex, including *C. deuterogattii*/VGII strains 14.1433, 93/980, and R265, C. *bacillisporus*/VGIII strain ATCC 32608, and *C. tetragattii*/VGIV strains MMRL2879, WM2872, and WM2876/V00869 (Table 1). They were not observed in any *C. gattii*/VGI strains. Figure 2D shows the maximum cell diameter observed among cells for the different strains in each species, with representative images of giant cells shown in Fig. 2E. Giant cells never formed more than 5% of the cells within a strain and produced regularly sized daughter cells.

### Stress tolerance testing indicates *C*. *deuterogattii*/VGII strains have elevated thermal tolerance, but there are no appreciable differences among species in tolerance to oxidative stress, salt, and detergent.

**FIG 3:**
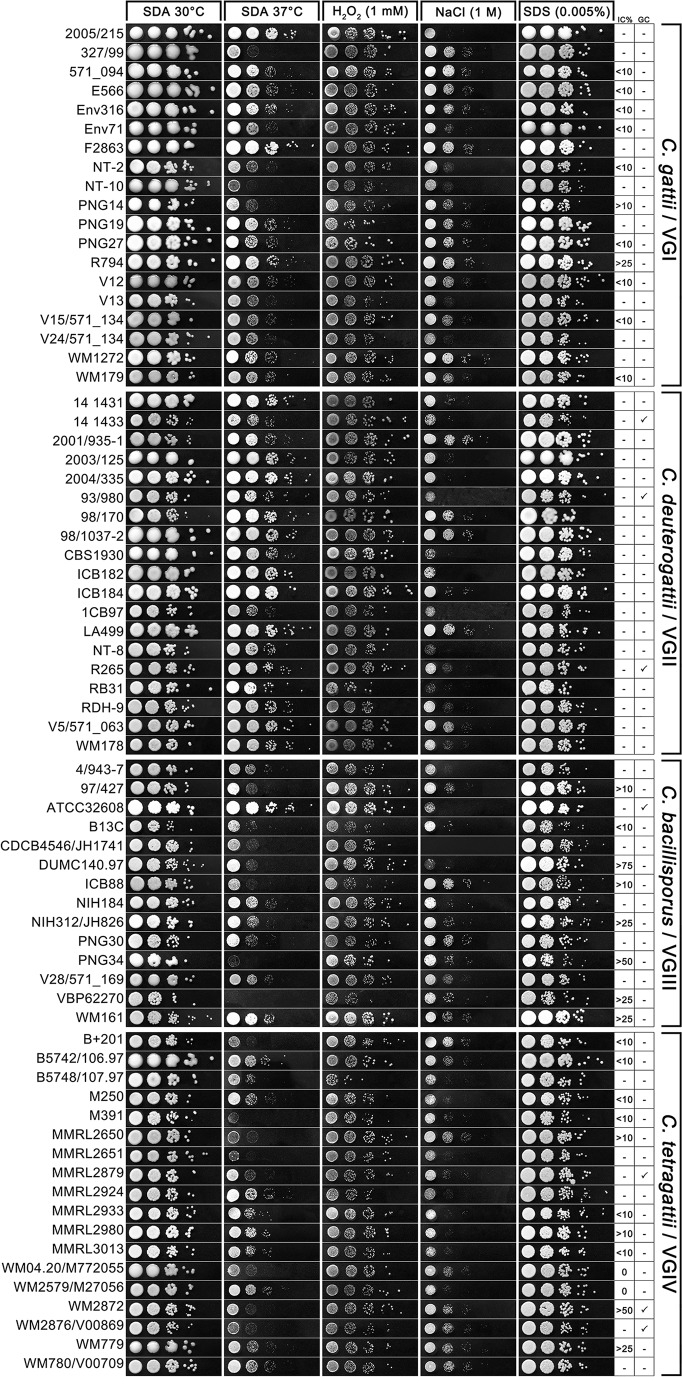
Stress tolerance spot plate assays of strains in the *C*. *gattii* species complex. Strains from each species were spotted in a 10-fold dilution series. For each strain, from left to right, the cryptococci are shown starting at 10^6^ cells/ml on unsupplemented SDA and incubated at 30°C and then shown grown on unsupplemented SDA and incubated at 37°C, and SDA supplemented with either 1 mM H_2_O_2_, 1 M NaCl, or 0.005% SDS and incubated at 30°C. Results were read after 48 h. The percentage of irregular cells (IC%) and presence (checkmark) or absence (−) of giant cells (GC) are shown to the right of the images.

Strains were subjected to growth at human body temperature (37°C) or with oxidative stress (1 mM H_2_O_2_) in order to test their tolerance to physiologically relevant stresses. Strains were also subjected to growth with salt (1 M NaCl) or detergent (0.005% SDS) to test whether irregular cells were more likely to occur in strains with defects in cell wall or membrane integrity. The growth of strains at 37°C varied among species (Fig. 3). Overall, *C. deuterogattii*/VGII exhibited the highest tolerance to 37°C with most strains showing growth levels similar to that of the 30°C control. *C. gattii*/VGI strains generally grew less well at 37°C than at 30°C with strains 327/99, NT-2, and NT-10 showing particularly limited growth. The majority of *C. bacillisporus*/VGIII and *C. tetragattii*/VGIV strains had substantially limited growth at 37°C, and almost complete inhibition was seen in strains PNG34, VPB62270, and M391, despite being originally isolated from clinical infections. All strains grew less well on plates containing 1 mM H_2_O_2_ than on the SDA control, and there was a similar level of inhibition among the different strains and species. On the whole, strains impaired in growth at 37°C still grew relatively well on H_2_O_2_, indicating that the inability to tolerate elevated temperature is not due to a general inability to tolerate physiological stress; one exception was *C. bacillisporus*/VGIII strain VBP62270, which grew poorly on both plates. There was substantial variation in tolerance to 1 M NaCl, and *C. gattii*/VGI strains appeared slightly more tolerant than the other species. All strains grew well on plates containing 0.005% SDS. There were no apparent correlations between the ability to grow in the presence of salt or detergent and the presence of irregularly shaped cells.

## DISCUSSION

The difficulty of defining species boundaries based on morphological characteristics in many fungi has made molecular phylogenetic analyses increasingly important for detecting genetically distinct groups, which may then be characterized as separate species (34). In 2002, in a pioneering study, Fisher et al. defined *Coccidioides posadasii* as a sister species to *Coccidioides immitis* based principally on the genealogical concordance of individual gene phylogenies (35). Other medically important fungi found to comprise multiple cryptic species include *Histoplasma capsulatum*, with six cryptic species (36), *Paracoccoides brasiliensis*, with four cryptic species (37), and *Blastomyces dermatitidis*, with two cryptic species (38). Often there are no known differences in morphology prior to delineation as separate phylogenetic species, and in some cases, these have emerged subsequently (34, 35, 39). Reevaluation of the characteristics of species within complexes, informed by the knowledge of their genetic differentiation, has the potential to lead to the discovery of previously unknown phenotypic differences, including ones that are clinically significant.

Given their recent elevation to species status, an understanding of virulence-related phenotypes among species of the *C. gattii* complex and determination of clinically relevant differences are of fundamental importance. In their paper defining the new species in the *C. gattii* complex, Hagen et al. (13) found little differentiation in physiological growth profiles on a large diversity of carbon and nitrogen compounds, and although melanization, phospholipase activity, and protease activity were highly variable among strains, this did not partition according to species. However, as these analyses were performed on 29 strains across the complex, they may have lacked power for statistical validation (13).

Table 2 summarizes the phenotypic differences that were found among species in the *C. gattii* complex. The current study found that capsule thickness and yeast cell diameter differed in a species-specific manner (Fig. 2A and B) when cells were grown on CIM-20 under inducing conditions. On average, *C. gattii*/VGI strains had significantly larger capsules than all other species, while *C. deuterogattii*/VGII strains had significantly larger yeast cell diameters than all other species. Similar recent studies have reported that *C. gattii*/VGI strains had thicker capsules than *C. deuterogattii*/VGII and *C. bacillisporus*/VGIII strains (assessed following 1 week of growth on Littman’s oxgall agar at both 25 and 37°C; *n* = 12) (13) and that *C. gattii*/VGI and *C. bacillisporus*/VGIII strains had significantly thicker capsules than *C. deuterogattii*/VGII strains (assessed following 24 h of growth in RPMI 1640 in 5% CO_2_ at 37°C; *n* = 44) (40).

**TABLE 2:** Summary of the phenotypic characteristics of each species

Phenotypic characteristic	Value for species/genotype^a^			
	*C. gattii*/VGI (*n* = 19)	*C. deuterogattii*/VGII (*n* = 19)	*C. bacillisporus*/VGIII (*n* = 14)	*C. tetragattii*/VGIV (*n* = 18)
Avg cell diameter (μm) (variance)	7.0 (0.9)	8.4 (2.5)	6.6 (1.0)	7.1 (1.8)
Avg capsule thickness (μm) (variance)	6.1 (3.3)	2.6 (0.5)	3.2 (2.7)	4.4 (2.0)
Irregular cells (% strains)	58	0	57	56
Giant cells (% strains)	0	16	7	17
Thermotolerance	Intermediate	High	Low	Low
Oxidative stress tolerance	High	High	High	High
Osmotic stress tolerance	Low	Low	Low	Low
Cell wall integrity	High	High	High	High

a The data for average cell diameter and average capsule thickness exclude data for strains where >10% of cells were irregular. For thermotolerance, oxidative stress tolerance, osmotic stress tolerance, and cell wall integrity, “High” indicates growth similar to no stress control on SDA at 30°C and “Low” indicates substantial growth inhibition.

These results suggest that capsule is stimulated or regulated differently among species in the *C. gattii* complex under nutrient-limiting conditions. Interestingly, there was an inverse relationship between capsule thickness and cell size in *C. gattii*/VGI and *C. deuterogattii*/VGII, while *C. bacillisporus*/VGIII and *C. tetragattii*/VGIV cells grouped between the two for both properties (Fig. 2C), indicating that cellular growth may be hindered by favoring capsule synthesis or vice versa. A considerable amount of heterogeneity in capsule thickness was also observed within *C. gattii*/VGI, *C. bacillisporus*/VGIII, and *C. tetragattii*/VGIV compared to *C. deuterogattii*/VGII, despite the latter exhibiting the greatest level of heterogeneity in cell diameter (Table 1). The high level of similarity in capsule thickness across *C. deuterogattii*/VGII strains was unexpected given that they were sourced from various continents and cover a diversity of multilocus sequence types (MLSTs) compared to the *C. gattii*/VGI, *C. bacillisporus*/VGIII, and *C. tetragattii*/VGIV strains, which are less geographically and genetically diverse (41).

The outbreak on Vancouver Island in Canada and studies conducted with animal models suggest that *C. deuterogattii*/VGII is a more virulent species (1, 41, 42). Finding *C. deuterogattii*/VGII strains to have less capsule than the other species was therefore somewhat unexpected, as capsule is thought to be the major virulence determinant of *Cryptococcus* (25). An *in vivo* study using the wax moth model organism *Galleria mellonella* conversely found that, although not statistically significant, *C*. *bacillisporus/*VGII strains had larger capsules than the other species/genotypes in the *C. gattii* complex did (43). This may be because during the progression of systematic cryptococcosis, the infecting organism must adapt to various conditions at different time points. Large capsule size has been shown to reduce phagocytosis (44), and therefore, initial infection may favor highly encapsulated cells. However, smaller particles may disseminate from the lungs and cross the blood-brain barrier more easily (45, 46), and as such, less capsule may be advantageous during dissemination and the later stages of infection. The observation that *C. neoformans* isolates from central nervous system samples generally possess smaller capsules than those from lung samples may reflect this (47). As such, it is unclear whether a large capsular phenotype enhances the overall virulence of strains, and it is likely that the relationship between cryptococcal capsule size and virulence varies throughout the course of infection. It is possible that discrepancies seen between virulence and capsule size are due to how infection is initiated and the site from which samples are obtained.

Yeast cells with diameters greater than 15 µm (excluding the capsule) are frequently reported in *C. neoformans*, where an increase in their presence has been reported to intensify virulence (48, 49), and it is speculated that their large size protects them from phagocytosis (50). Few studies have noted the presence of these cells in *C. gattii*, although a recent study of infection in *G. mellonella* larvae observed cells with diameters ranging from 15 to 75 μm (43). Here we report the presence of giant cells in strains of *C. deuterogattii*/VGII, *C. bacillisporus*/VGIII, and *C. tetragattii*/VGIV (Table 1; Fig. 2D and E). However, it should be noted that these giant cells are not necessarily equivalent to titan cells, and giant cells observed in the current study were much smaller than the titan cells seen *in vivo*. Currently, no method exists to reliably and consistently induce giant or titan cells *in vitro* (33), and further work is needed to study this unique subpopulation of cells.

Substantial variation in thermotolerance was seen among the *C. gattii* complex species (Fig. 3). The ability to grow at 37°C is essential to successfully establish infection in humans and is a significant virulence factor (51), and it was therefore surprising that the majority of *C. bacillisporus*/VGIII and *C. tetragattii*/VGIV strains were substantially reduced in growth, with clinical isolates PNG34, VPB62270, and M391 almost completely inhibited. *C. deuterogattii*/VGII strains were the most temperature tolerant, suggesting they may have a greater ability to sustain infection in a mammalian host. Lower thermotolerance of *C. bacillisporus*/VGIII strains was also noted by Hagen et al. who found no strains of this species able to grow at 40°C, while strains from all other species, including *C. tetragattii*/VGIV could, although the amount of growth was not assessed (13).

The ability of pathogenic fungi to tolerate human physiological temperature is linked to the production of calcineurin, a calcium-dependent protein phosphatase (52, 53). *C. deuterogattii*/VGII strains have a higher tolerance of calcineurin inhibitors compared to other species in the *C. gattii* complex, and this may explain their observed thermotolerance (52). Calcineurin deletion mutants also have disruptions to their plasma membrane, and interestingly, they have morphological irregularities similar to those observed here for some of the *C. gattii*/VGI, *C. bacillisporus*/VGIII, and *C. tetragattii*/VGIV strains (Fig. 2B). However, calcineurin deletion mutants are highly sensitive to the potent cell wall stressor SDS (54), which was not seen for strains with morphological irregularities in the current study. Likewise, some variation in tolerance to 1 M NaCl did not correlate with the presence of irregular cells, indicating that these did not possess substantial defects in cell wall or membrane integrity. Phenotypic plasticity and pleomorphism, which are reported in various pathogenic yeasts, including *Cryptococcus* species as mechanisms for rapid adaptation (55, 56), could explain this irregular phenotype. They are considered to be important for stress tolerance and response to environmental cues, which can in turn result in changes to virulence in the host environment.

### Concluding remarks.

Significant differences in cell size, capsule size, and thermotolerance were observed among species in the *C. gattii* complex, which may in part reflect their differing epidemiology and ability to cause outbreaks. These results confirm significant *in vitro* phenotypic variation among the newly described species and that there is a complex relationship between capsule size and adaptation to the host environment. A limitation of this study is that it used *in vitro* conditions to simulate host stress, and exactly how the differences reported here play out in mammalian infection remain to be elucidated. Future work using cell culture-based methods and animal models will help to gain a better understanding of the relationship between capsule and other virulence attributes in mammalian hosts across the *C. gattii* species complex.

## MATERIALS AND METHODS

### Species and strain selection.

As the use of the new species names is yet to be fully accepted, we use both the new species designations and the molecular genotype in this study and refer to them collectively as the *C. gattii* species complex. Seventy strains within the *C. gattii* species complex were used in this study, with details listed in Table 1. Genetically and geographically diverse strains of each species were targeted based on data compiled by Campbell et al. (57), Carter et al. (58), and Fraser et al. (41).

### Growth conditions and preparation of strains.

Strains were cultured from −80°C glycerol stocks, streaked for single colonies on Sabouraud dextrose agar (SDA; 10 g peptone, 40 g glucose, 15 g agar, 1 liter distilled H_2_O [dH_2_O]) and incubated at 30°C for 48 h. To standardize the growth phase for each strain, strains were grown overnight from single colonies in 50 ml of yeast nutrient broth (YNB; 34.53 g MOPS, 6.7 g yeast nitrogen base, 5 g glucose, 1 liter dH_2_O) in a 100-ml Schott bottle at 37°C with shaking (180 rpm) until they reached exponential growth phase.

### Capsule induction.

Capsule induction was first optimized using various different media based on those used to induce capsule in *C. neoformans* (27, 59). These media included Dulbecco’s modified Eagle medium (DMEM; Life Technologies, Inc.) and SDA diluted 10-fold (CIM-10) (27), 20-fold (CIM-20), or 50-fold (CIM-50) in 50 mM morpholinepropanesulfonic acid (MOPS) (Sigma-Aldrich). A single loopful of cells was taken from the overnight culture and streaked for single colonies onto each medium. The plates were incubated with 5% CO_2_ at 37°C for 5 days. For a control, strains were streaked for single colonies on SDA and incubated at 30°C for 5 days.

### India ink staining and microscopy.

To visualize capsule, single colonies were suspended in 150 μl of phosphate-buffered saline (PBS) (Oxoid) and counterstained with 20 μl of India ink (Pébéo). A 15-µl aliquot of this mixture was placed on a glass slide and dried for 10 min under a coverslip. The slides were then photographed using an IS10000 inverted microscope (Luminoptic) under the 40× objective using ISCapture Imaging software (Tucsen Photonics). For each strain, a minimum of four random fields of view were photographed.

### Measurement of cell and capsule size.

Total diameter (including capsule) (*d*_*t*_) and yeast cell diameter (*d*_*y*_) were measured for 50 cells per strain using ImageJ software (National Institutes of Health). From these measurements, capsule thickness (*t*_*c*_) was calculated as $\frac{1}{2(d_{t}-d_{y})}$. Total volume (*v*_*t*_) and yeast cell volume (*v*_*y*_) were calculated using the formula for the volume of a sphere $\left(\frac{4}{3}\pi r_{d}{}^{3}\right)$. Morphologically irregular cells were excluded from measurements, and strains where >10% of cells had morphological irregularities were not measured.

### Plate spotting assays.

Exponentially growing overnight cultures were adjusted to a concentration of 10^6^ CFU/ml, and serial 10-fold dilutions were prepared in sterile PBS. A 5-µl aliquot of each dilution was spotted onto SDA plates supplemented with 1 mM H_2_O_2_, 1 M NaCl, or 0.005% SDS to assess oxidative stress tolerance, salt tolerance, and cell well integrity, respectively. Growth at 37°C was assessed on SDA plates with no supplements. All plates were incubated at 30°C unless otherwise specified and photographed after 48 h. Images were assembled and contrast adjusted using Photoshop CS5 (Adobe Systems).

### Statistical analysis.

Statistical significance was calculated using two-tailed unpaired *t* tests with Welch’s correction. Differences in variance between species/genotypes were assessed by F test analysis. *P* values of <0.05 were considered significant. Error bars represent the means ± 95% confidence intervals. All data were analyzed using Excel (Microsoft Corporation) and Prism 5 (GraphPad Inc.) software.
